# False Impressions

**Published:** 1888-10-27

**Authors:** Caroline Fothergill

**Affiliations:** Author of "An Enthusiast," "A Voice in the Wilderness," etc.


### FALSE IMPRESSIONS.

By Caroline Fothergill, Author of "An Enthusiast," "A Voice in the Wilderness," etc.

(*Continued from p.* 46.)

### Chapter IV.—Meeting the Third.

About a fortnight after the events narrated in the last chapter Beatrice was summoned to a committee meeting at the Murray Museum. Laura had once asked her why it was called the Murray Society, and Beatrice answered that she had been told the man who had been chiefly instrumental in getting up the society was called Murray, but further than that, or who Mr. Murray was, she was herself ignorant. She had arranged her own engagements so that she should always be free on committee afternoons, and she had determined to give as much time as she could to the work. She already felt attached to the place, and experienced quite a glow of feeling as she walked up the smoky enclosure, by courtesy called a garden, in front of the house, and entered the building. She passed lightly up the staircase, casting quick glances at the pictures which lined the walls on either side, and at the life-size Apollo who mounted guard at the head of the stairs. She felt gay and light of heart as she trod the corridor leading to the committee-room, pausing from time to time to refresh herself with a sight of some of her favourite works of art. She heard a buzz of voices coming from the room to which she was going. The committee had assembled in force, for the winter programme was in course of preparation, and after the dead summer season people were settling down to work again with vigour. Beatrice greeted them all; by this time she knew nearly every member of the committee, some of whom she had not seen for some time. Tea was brought in, and work began.

Beatrice, in all the ardour of a beginner, was feeling that she could do gre t things, and had promised to undertake the Thursday evening readings to children for the first three months of the season, and also to work up several "entertainments." These entertainments were held twice a week, and were very popular, consisting of songs, recitations, and instrumental music. She was pledging herself right and left to the secretary, and other conversation was going on among other people, when someone said—

"By the way, where is Murray? He ought to have been here. Does anyone know if he has come home? Has anyone seen him?

"Yes," said the secretary, looking up from the note-book in which he was busily writing, "he came home a month ago, and he will be here this afternoon. He has brought a lot of things from America. I can't tell why he is so late."

Beatrice heard all this, and thought within herself, with some satisfaction "I am going to see Mr. Murray at last." She turned to a lady sitting near her and said—

"I think I have never met Mr. Murray."

"No, he has been away since before you joined us."

"I suppose he is an important person here."

"Oh, he is, we could not get on without him. He holds the threads of the whole affair, and comes here nearly every day, I think."

Just then, someone was heard coming along the corridor outside; the step was light and firm, that of a young man, and one or two people exclaimed simultaneously—

"Here he is, this is Murray."

Beatrice had just time to think, "he seems very popular, they evidently all like him," when the step stopped at the door, a key was fitted into the lock, the door opened, and Beatrice, looking up with everyone else, found her eyes resting upon the familiar, though only twice seen, figure of her "ghost."

She did not blush this time; instead, she went very pale, and although she at once lowered her eyes, she was conscious that he had seen and was looking at her. There was a bustle of greeting and welcome, during which she sat pale and silent, as yet too overwhelmed to make any effort to shake off the strange feeling which oppressed her. The question of where and when they would meet again was conclusively answered. Then someone spoke to her, and she heard her name and Murray's spoken in quick succession. They were evidently being introduced, and she bowed slightly, and received an equally slight inclination of the head from him. After that, he turned away without speaking, accepted a cup of tea, and entered into conversation with the secretary, leaving Beatrice with a feeling of having been snubbed which she had never experienced before.

This did not last long. Her natural spirit reasserted itself. She was seated a little apart from the others; now she approached them again, and began to join in the conversation.

Although she never looked directly at Murray, she was intensely conscious of his presence in the room, and she was also aware that he never looked at her, neither did he address her, even when once he was standing close by her, and neither was speaking to anyone else. He was treating her with a negligence to which she was not at all accustomed, and her spirit rose under such treatment. What had she done to deserve it? And even had she been guilty of any fault, it was extremely impertinent of him to behave as he was doing. He could not be less drawn towards her than she was towards him, and in public, at least, they might keep up the forms of ordinary politeness. Her imperious temper took fire at his manner, and, obeying a momentary impulse, she turned to him and said—

"I suppose you take a very active share in this work."

"Yes," he answered, without changing countenance at all, "I am very much interested in it, and my time is my own."

She said nothing more, and there was a short silence until he went on in the same quiet, even, perfectly courteous tone—

"You are probably new to this kind of thing. I think you have joined the committee during my absence; do not let our zealous secretary persuade you to do too much. He tells me you have already promised a good deal."

"I have promised to take some of the readings to children," she answered in a tone as unemotional as his own.

"Ah!" he said, just glancing at her, "that is very kind of you. It is particularly important to civilise the children; but I had better give you a caution. Many of these children come from the poorest and lowest parts of the town, and some people are afraid of infection. I do not for an instant suppose there is the slightest danger, but a lady who gave some reading here last winter was laid up with diphtheria some months after, and," with a sarcastic smile curling his lips, "she has withdrawn her name from our list of helpers this year. It does not do to be nervous, for your own sake and for ours too. It is very inconvenient to have help which had been promised suddenly withdrawn."

Through all this long speech his voice had never wavered from its tone of calm courtesy, there was not a flaw in his way of saying it all, but what he said made Beatrice's colour rise, and she bit her lip with anger. She looked straight at him, and her voice was perfectly calm as she said—

"It is considerate of you to warn me, but I am not afraid. I am not naturally nervous."

"Ah!" he answered, "that is very fortunate. So many ladies are, but the case I have mentioned was the only one of its kind, and the lady was perhaps exceptionally nervous."

"Oh! I quite appreciate your motive in speaking," said Beatrice with a smile, upon which he also smiled, and bowed, and walked away; and she, looking at her watch, discovered that it was time to go home.

She walked home, thinking that in that way she might walk off some of her anger and annoyance. They had met and become acquainted without any effort on the part of either, and so far their acquaintance had not been pleasant. She rebelled against his manner with all the force of a strong will which suddenly finds circumstances removed beyond its control or guidance. For a time she felt as if this strange acquaintance, with its background and setting, was beyond her powers of endurance, and she thought of withdrawing from this work, and so avoiding any further intercourse with Mr. Murray; but the next instant she felt that to do so would be childish in the extreme, and would only amuse him. So she decided, for the present at any rate, to let things remain as they were. She realised, however, that the gilt had been suddenly and ruthlessly rubbed off this new social interest. It was very annoying. She had looked forward to this work so much, and had taken it up rather against Laura's wishes, for Mrs. Ledward thought the whole scheme ridiculous and unpractical, and was never weary of prophesying that only evil would come of Beatrice mixing herself up in it.

She debated in her mind as to whether she should tell

Laura of what had happened, and finally decided that she would. Her circle of acquaintance was constantly extending; it might before long be inevitable that Mr. Murray was included in it, elsewhere than in his own museum. To keep Laura in the dark might have awkward results; otherwise, the whole subject was distasteful to her, and she would willingly never have spoken of it to anyone. So, as they sat together in the evening, she said abruptly—

"I have seen my ghost again to-day."

"No!" said Laura with excitement. "Where? Have you discovered who he is? Tell me about him; unravel the mystery, I beg."

"There is no mystery to unravel. He belongs to the Murray Committee, and was there to-day for the first time since I joined."

"How interesting! And what may be his noble name?"

"Murray—George Murray. It is after him that the whole affair is named, he is the guiding spirit of it all."

"Very curious indeed!" said Laura, nodding her head. Were you introduced?"

"Yes. So the mystery is now at an end."

"Nothing of the kind, far from it. Where did you first see him? It was not at Mrs. Lennox's."

Breatrice hesitated, and then resolved to make a clean breast of it.

"You remember my visit to Dr. Temple at Clearwater, and my saying there was a gentleman waiting to see him as well as myself?"

"And he is the man?" asked Laura, clasping her little jewelled hands in excitement.

"Yes, the same."

"How curious! And what is he like to talk to?"

"Very disagreeable indeed."

"I am sorry for that. It is curious too, people are not usually disagreeable in talking to you;" and her eyes, full of both admiration and affection, sought her friend's beautiful face.

"You see," said Beatrice with some hesitation, and there stopped.

"I don't," said Laura energetically—"I don't see. That is just what I complain of, I am not allowed to see anything."

"Well, I have not felt inclined to talk about it," said Beatrice, with a slight tone of apology in her voice. "However, I will tell you now if you care to know," and she forthwith related all with which we are already acquainted, and to which Laura listened with the deep interest she always felt in anything concerning Beatrice. Miss Stanford concluded with—

"Of course he thinks I was telling an untruth."

"It is awkward," murmured Laura reflectively. "What shall you do?"

"I don't know. I should like him to know that he was mistaken. If I can find an opportunity I think I shall explain matters."

"Shall you?" dubiously. "I don't think I should. If I were you I should let it alone."

"I can't let it alone!" colouring as she spoke. "The situation will become unbearable if I let it alone. Besides, I do not wish him to think of me unjustly."

Laura looked astonished.

"What does it matter what he thinks," she said, raising her eyebrows. "He is practically a stranger, and it will be perfectly easy to keep him at arm's length; you excel in doing that you know. You don't generally care so much about the opinion of other people"

Beatrice turned her head aside, perhaps to hide her rising colour.

"I can't keep him at arm's length," she said at last. "He is the person down there; we shall be constantly meeting, and having to work together. We must appear friendly."

"Well, leave it to him," said Laura bluntly, but with excellent sense. "It is for him to act; why trouble yourself about it? Let him ask for an explanation if he wants one, and if he does not, leave it alone. Don't get morbid about it."

Beatrice was silent. She could not have made Laura understand what was by no means clear to herself, and she only said—

"Then you would leave it alone?"

"I should, but I would not presume to advise you, and I know just how you will feel. If you don't have the explanation you will wish you had done, and if you do you will wish you hadn't." And with this sibylline utterance she returned to her book.

*(To be continued.)*

